# Activated prothrombin complex concentrate in patients receiving emicizumab prophylaxis: from evidence to clinical practice

**DOI:** 10.1016/j.rpth.2025.102926

**Published:** 2025-06-17

**Authors:** Robert F. Sidonio, Guy Young, Carmen Escuriola Ettingshausen, Johnny Mahlangu, Margareth C. Ozelo, Alok Srivastava, Jerzy Windyga, Hye–Youn Lee, Aurelia Lelli, Steven W. Pipe

**Affiliations:** 1 Hemophilia of Georgia Center for Bleeding and Clotting Disorders of CHOA, Atlanta, Georgia, USA; 2 Cancer and Blood Disorders Institute, Children’s Hospital Los Angeles, University of Southern California Keck School of Medicine, Los Angeles, California, USA; 3 Hämophilie Zentrum Rhein Main (HZRM), Frankfurt, Germany; 4 Department of Molecular Medicine and Haematology, School of Pathology, Faculty of Health Sciences, University of the Witwatersrand and National Health Laboratory Service, Johannesburg, Gauteng, South Africa; 5 Hemocentro University of Campinas, Department of Internal Medicine, School of Medical Sciences, University of Campinas, Campinas, São Paulo, Brazil; 6 Haematology Research Unit, St. John’s Research Institute and Department of Clinical Haematology, St. John’s Medical College Hospital, Bengaluru, India; 7 Department of Hemostasis Disorders and Internal Medicine, Laboratory of Hemostasis and Metabolic Diseases, Institute of Hematology and Transfusion Medicine, Warsaw, Poland; 8 Takeda Pharmaceuticals International AG, Zurich, Switzerland; 9 Departments of Pediatrics and Pathology, University of Michigan, Ann Arbor, Michigan, USA

**Keywords:** activated prothrombin complex concentrate, breakthrough bleeding, bypassing agents, congenital hemophilia A with inhibitors, emicizumab, nonfactor therapies

## Abstract

Nonfactor therapies (NFTs) such as emicizumab are increasingly becoming established for bleed prophylaxis in people with hemophilia A. Both classes of bypassing agents (BPAs; activated prothrombin complex concentrate [aPCC] and recombinant activated factor [F]VII [rFVIIa]) are required for effective bleed management in people with inhibitors receiving NFT prophylaxis, owing to the variable hemostatic responses to aPCC and rFVIIa seen in different patients. Early reports of rare thrombotic events in clinical trials of emicizumab led to the development of guidelines preferring the use of rFVIIa over aPCC for the management of breakthrough bleeding and major surgery in patients receiving emicizumab. Since these guidelines were issued, data from clinical and real-world studies have emerged that support the efficacy and safety of aPCC in this setting. In this narrative review, we aimed to evaluate the evidence on bleed management with aPCC in people with hemophilia A with inhibitors receiving emicizumab prophylaxis, as well as other NFTs. These emerging data indicate that aPCC used at doses of ≤ 100 U/kg/day in people with inhibitors receiving emicizumab is not associated with thrombotic microangiopathy or thrombotic events. As more NFTs become available for bleed prophylaxis, both classes of BPAs will be needed for bleed management in people with inhibitors. Thus, optimal use of BPAs is critical and will require an understanding of the factors that influence BPA selection, such as bleed type and location, clinical response, dosing and duration of use, accessibility and availability, and patient or caregiver preference.

## Essentials

• People with hemophilia A receiving NFTs experience breakthrough bleeding.
• Both classes of BPAs are needed for bleed management in people with inhibitors.
• This review summarizes data on the use of aPCC in people receiving emicizumab prophylaxis.
• Evidence supports the use of aPCC at doses of ≤ 100 U/kg/day in people receiving emicizumab.

## 1 Introduction

In people with hemophilia A, replacement therapy with factor (F)VIII concentrates has been the standard of care for the treatment of acute bleeding episodes and surgical prophylaxis [1,2]. However, about 30% of patients develop alloantibodies (inhibitors) that neutralize the hemostatic function of these clotting factor concentrates [2]. Bleed prevention and treatment in people with inhibitors is more challenging than in those without inhibitors, and inhibitor development is associated with an increased disease burden and higher morbidity and mortality [2]. Bypassing agents (BPAs; activated prothrombin complex concentrate [aPCC] and recombinant activated FVII [rFVIIa]) can be used for the treatment of acute bleeding in people with hemophilia A with inhibitors, because their mechanisms of action circumvent the need for FVIII to generate thrombin and achieve hemostasis [2].

Nonfactor therapies (NFTs) are increasingly becoming the standard of care for bleed prophylaxis in people with hemophilia A [2,3]. They differ from conventional prophylactic treatments in that they do not replace the missing FVIII in these patients [2]. Instead, their modes of action include mimicking the bridging function of activated FVIII (FVIIIa) or inhibiting natural anticoagulants, such as tissue factor pathway inhibitor (TFPI) or antithrombin, to rebalance hemostasis [4]. At the time of writing, NFTs indicated for prophylaxis in people with hemophilia A with or without inhibitors are the FVIIIa mimetic emicizumab, which is the most studied and widely available NFT, and the rebalancing therapies concizumab and marstacimab, which target TFPI, and fitusiran, which targets antithrombin [2,[5], [6], [7], [8], [9], [10], [11], [12]]. Nevertheless, in people with inhibitors receiving NFT prophylaxis, BPAs are still required for the management of breakthrough bleeding or major surgeries [2,[13], [14], [15], [16], [17], [18]]. In patients receiving emicizumab prophylaxis, BPA treatment may be considered as a cotreatment, as residual plasma levels of emicizumab remain for up to 6 months after therapy discontinuation [13]. There have been no clinical studies primarily designed to investigate the efficacy and safety of either class of BPAs in this context, with evidence instead arising from studies that focused on the efficacy and safety of NFTs [15,[18], [19], [20], [21], [22], [23]]. Thus, there is a need for an in-depth examination of the available evidence on the efficacy and safety of BPAs in patients receiving NFT prophylaxis.

Thrombotic events (TEs) are rare in patients receiving emicizumab and BPAs as standalone therapies or when combined [[20], [21], [22],[24], [25], [26], [27], [28]], although 3 thrombotic microangiopathies (TMAs) and 2 TEs were reported in 5 people with hemophilia A with inhibitors who were receiving emicizumab prophylaxis and were treated with BPAs for breakthrough bleeding in the HAVEN 1 clinical trial [20]. Two of the patients who experienced TMAs were treated with both rFVIIa (eptacog alfa) and aPCC (> 100 U/kg/day for > 48 hours); the third patient received only aPCC (> 100 U/kg/day for > 48 hours) [20,22,29,30]. The 2 patients who experienced TEs were treated with aPCC (> 100 U/kg/day for 24-48 and > 48 hours, respectively). Importantly, no TMAs or TEs were observed in any of the patients who received aPCC at a dose of ≤ 100 U/kg/day [20]. However, the occurrence of the 3 TMAs in HAVEN 1 led to a caution against the use of aPCC at a cumulative dose of > 100 U/kg/day for > 24 hours in the US product label for emicizumab and resulted in guidelines preferring rFVIIa over aPCC for the management of breakthrough bleeding and major surgery in patients receiving emicizumab [2,20,22]. Subsequently, the use of aPCC in patients receiving emicizumab has reduced, with rFVIIa often being the only treatment option available for acute hemostatic management in these patients [31]. This is suboptimal for both patients and their physicians, because it limits individualized therapeutic approaches to bleed management [2,31].

Both classes of BPAs are required for optimized bleed management in people with hemophilia A with inhibitors, owing to the variable hemostatic responses to aPCC and rFVIIa seen in different patients [2,31,32]. Substantial data are now available on the use of aPCC and emicizumab from both clinical trials and real-world evidence (RWE), which are critical for informing the medical community on how aPCC can be used safely in the management of breakthrough bleeding and major surgery in patients receiving emicizumab prophylaxis.

In this narrative review, we aimed to evaluate the available evidence on the use of aPCC in people with hemophilia A with inhibitors receiving emicizumab prophylaxis, as well as other NFTs, and discuss the implications of these data for clinical management.

## 2 Breakthrough Bleeding in People With Hemophilia A With Inhibitors Receiving Emicizumab Prophylaxis

People with hemophilia A with inhibitors receiving prophylaxis with emicizumab experience breakthrough bleeding, which requires treatment with BPAs (aPCC or rFVIIa) to achieve hemostasis [2]. Most of these bleeds occur in the joints, with muscles as the second most common bleed site [19]. Prompt treatment of all bleeds is essential because even a single bleed can lead to irreversible damage [2,33]. Despite this, a high proportion of bleeds remain untreated, with untreated bleeds observed more frequently in people with inhibitors than in those without inhibitors [19]; the long-term implications of this are unknown. Importantly, studies typically do not report whether tranexamic acid was used in association with treated or untreated bleeds. Moreover, there is considerable variation in the incidences of breakthrough bleeding (both treated and untreated) reported across emicizumab studies. In a comprehensive review by Young et al. [3], the median proportion of emicizumab-treated people with hemophilia A (with or without inhibitors) who experienced treated bleeds was 37% in clinical trials and 30% in RWE studies.

### 2.1 Clinical trial data

A summary of breakthrough bleeding rates in people with hemophilia A with inhibitors receiving emicizumab prophylaxis in clinical trials is shown in Table 1. In the HAVEN 1 and 2 clinical trials, breakthrough bleeding (all bleeds) was reported in 37% (13/35) and 51% (33/65) of patients, respectively [20,21]. In the STASEY study, rates of breakthrough bleeding were 45% (88/195) for all bleeds and 17% (34/195) for treated bleeds [26]. The HAVEN 4 trial investigated emicizumab prophylaxis in a combined population of people with and without inhibitors, for whom rates of breakthrough bleeding were 71% (29/41) for all bleeds and 44% (18/41) for treated bleeds [23].

**Table 1:** Rates of breakthrough bleeding in people with hemophilia A with inhibitors receiving emicizumab prophylaxis in clinical trials.

Study	Patient population^a^	Patients with treated bleeds, *n*/*N* (%)^b^	Patients with any bleeds, *n*/*N* (%)^b^
HAVEN 1 (NCT02622321) [20]	HA with inhibitors^c^	NR	13/35 (37)
HAVEN 2 (NCT02795767) [21]	HA with inhibitors^d^	15/65 (23)	33/65 (51)
STASEY (NCT03191799) [26]	HA with inhibitors	34/195 (17)	88/195 (45)
HAVEN 4 (NCT03020160) [23]	HA with or without inhibitors	18/41 (44)	29/41 (71)

HA, hemophilia A; n, number of patients who did not experience (treated or any) zero bleeds; N, number of patients in the study-specific population; NR, not reported.

a Patients were adults and adolescents (aged ≥ 12 years), with the exception of HAVEN 2, which enrolled pediatric patients (aged < 12 years). In HAVEN 4, data for people with HA with inhibitors were reported together with those without inhibitors.

b Rates of breakthrough bleeding were calculated from the number of patients in the study-specific population who received emicizumab prophylaxis and did not experience zero bleeds (treated or any, respectively).

c Patients who were randomly assigned to receive emicizumab prophylaxis (group A).

d Patients who received emicizumab prophylaxis once weekly (group A).

An analysis of patients who were enrolled in HAVEN 1 or 2 after receiving prophylaxis with BPAs in a noninterventional study found that 44% of bleeds (27/61) in adult and adolescent patients and 93% of bleeds (83/89) in pediatric patients were untreated during the clinical trials [19]. The most common locations for treated bleeds in the adults and adolescents were joints (71% [706/997]) and muscles (15% [152/997]). Joint bleeds were also the most common type of untreated bleeds (55% [362/659]), followed by bruise/hematoma bleeds (16% [104/659]) and muscle bleeds (14% [89/659]) [19].

### 2.2 RWE

A summary of breakthrough bleeding rates in real-world studies of people with hemophilia A with inhibitors receiving emicizumab prophylaxis is shown in Table 2 [[34], [35], [36], [37], [38], [39], [40], [41]]. Incidences of breakthrough bleeding reported in Asia include 43% of people with or without inhibitors (13/30; all bleeds) at the hemophilia treatment center of a tertiary care hospital in Bangladesh [34] and 49% (19/39), 44% (16/36), and 19% (5/26) of people with inhibitors (treated bleeds) in the first, second, and third years of emicizumab prophylaxis, respectively, at 17 hemophilia care centers in Taiwan [35].

**Table 2:** Real-world rates of breakthrough bleeding in people with hemophilia A with inhibitors receiving emicizumab prophylaxis.

Country	Patient population^a^	Emicizumab dosing	Patients with bleeds, *n*/*N* (%)
Bangladesh [34]	HA with or without inhibitors	6 mg/kg every 4 wk	13/30 (43)
Taiwan [35]	HA with inhibitors	1.5 mg/kg every wk (*n* = 19)^b^ 3.0 mg/kg every 2 wk (*n* = 16) 6.0 mg/kg every 4 wk (*n* = 4)	Y 1 (treated bleeds): 19/39 (49) Y 2 (treated bleeds): 16/36 (44) Y 3 (treated bleeds): 5/26 (19)
Canada [36]	HA with inhibitors	Every wk, every 2 wk or other	10/49 (20)
UK [37]	HA with inhibitors	Every wk (*n* = 57) Every 2 wk (*n* = 58) Every 4 wk (*n* = 2)	All patients (treated bleeds): 13/117 (11) • Children (aged < 12 y): 1/40 (3) • Adolescents (aged 12-18 y): 3/12 (25) • Adults (aged > 18 y): 9/65 (14)
USA [38]	HA with inhibitors in the first 6 mo of emicizumab prophylaxis	Every wk (*n* = 12) Every 2 wk (*n* = 7)	1/19 (5)
USA [39]	HA with inhibitors	1.5 mg/kg every wk	8/24 (33)
Israel [40]	HA with or without inhibitors	1.5 mg/kg every wk	Children (aged ≤ 18 y): 29/58 (50) Adults (aged ≥ 19 y): 25/49 (51)
Israel [41]	HA with or without inhibitors who had received emicizumab for ≥ 18 mo	1.5 mg/kg every wk or 3.0 mg/kg every 2 wk	Traumatic bleeds: 43/70 (61) Spontaneous bleeds: 36/70 (51)

HA, hemophilia A; mo, month; n, number of patients who received a specific emicizumab dosing regimen [emicizumab dosing column], or did not experience (treated or any) zero bleeds [patients with bleeds column]; N, number of patients in the study-specific population; wk, week; y, year.

a Patients of any age were eligible for inclusion in the studies. Duration of emicizumab treatment and data for specific age groups are shown where available. In some studies, data for people with HA with inhibitors were reported together with patients without inhibitors, as indicated.

b Of the 19 patients, 15 who initially received a weekly dose were switched to every-2-week dosing.

In Western countries, reports of breakthrough bleeding have been captured in national registries, databases, and dedicated hemophilia treatment centers. The Canadian Bleeding Disorders Registry (data captured up to December 31, 2020) reported breakthrough bleeds in 20% of emicizumab-treated people with hemophilia A with inhibitors (10/49) [36]. In the UK National Haemophilia Database (data captured from January 1, 2018, to September 30, 2021), 11% of people with hemophilia A with inhibitors receiving emicizumab (13/117) were reported to have experienced treated breakthrough bleeding episodes [37]. Three US hemophilia treatment centers reported an incidence of breakthrough bleeding of 5% (1/19) in the first 6 months of emicizumab prophylaxis in a small cohort of people with hemophilia A with inhibitors [38]. At another hemophilia treatment center in the US, 33% of people with hemophilia A with inhibitors (8/24) experienced breakthrough bleeding episodes (data captured from November 1, 2017, to May 31, 2019) [39].

The Israeli National Hemophilia Center has also reported breakthrough bleeding in people with hemophilia A with or without inhibitors initiating emicizumab prophylaxis (51% [25/49]) [40] and in those who have received emicizumab for ≥ 18 months (61% [43/70] experienced traumatic bleeding; 51% [36/70] experienced spontaneous bleeding) [41].

## 3 Efficacy and Safety of aPCC in Patients Receiving Emicizumab Prophylaxis

A summary of data on the use of aPCC in people with hemophilia A with inhibitors receiving emicizumab prophylaxis is shown in Table 3 [14,22,26,39,[42], [43], [44], [45], [46]]. In 152 instances of aPCC treatment in 50 patients receiving emicizumab prophylaxis, 2 TEs and 5 TMAs were reported, all of which occurred in patients who received aPCC at doses of > 100 U/kg/day for ≥ 24 hours; 4 of the 5 TMAs occurred in patients who were also treated with rFVIIa for the same bleeding event.

**Table 3:** Use of activated prothrombin complex concentrate in people with hemophilia A with inhibitors receiving emicizumab prophylaxis.

Study	No. of patients and bleeding episodes/surgeries managed with aPCC	Dosing summary and thrombotic complications
Breakthrough bleeding		
Pooled analysis of **HAVEN 1-4** [22]	130 bleeds in 37 patients	• Bleeds treated with a cumulative dose of aPCC ≤ 100 U/kg/day: 99/130 (76%) o Bleeds treated for ≤ 24 h: 87/130 (67%) o Bleeds treated for 24-48 h: 6/130 (5%) o Bleeds treated for > 48 h: 6/130 (5%) • Bleeds treated with a cumulative dose of aPCC > 100 U/kg/day: 31/130 (24%) o Bleeds treated for ≤ 24 h: 18/130 (14%) o Bleeds treated for 24-48 h: 3/130 (2%) o Bleeds treated for > 48 h: 10/130 (8%) • Median (minimum to maximum) number of aPCC infusions in HAVEN 1: 3 (1-27) [20] • **3 TM** **A** **s reported** a o All treated with aPCC > 100 U/kg/day for > 48 h o 2/3 treated with rFVIIa for same bleeding event [20] • **2 TEs reported** o Both treated with aPCC > 100 U/kg/day for > 24 h (1 for > 24 h; 1 for > 48 h)
**STASEY** [26]	13 bleeds in 5 patients	• Median cumulative dose of aPCC per bleed: 10.9 U/kg • Median (minimum to maximum) number of infusions: 1 (1-2) • Bleeds treated with a single infusion: 8/13 (62%) • Bleeds treated with 2 infusions: 5/13 (38%) • **No TMAs or TEs reported**
Zimowski et al. [42]	3 bleeds in 2 patients • Traumatic skull fracture with extracranial hematoma • Traumatic facial hematoma • Spontaneous mesenteric hematoma with hemoperitoneum	• Lack of response with rFVIIa 80-90 μg/kg every 3-12 h (escalating frequency) for 6-10 d • Switched to aPCC 10-15 U/kg every 12-24 h for 5-10 d • **No TMAs or TEs reported**
Batsuli et al. [43]	1 bleed in 1 patient • Spontaneous iliopsoas hematoma	• Lack of response with rFVIIa 225 μg/kg (initial dose), followed by rFVIIa 75 μg/kg every 6 h for 24 h • Switched to aPCC 25-50 U/kg every 8-12 h for 18 d • **No TMAs or TEs reported**
Dargaud et al. [44]^b^	1 bleed in 1 patient • Perirenal hematoma	• aPCC 25 U/kg (initial dose), followed by aPCC 15 U/kg every 12 h for 2 wk, then aPCC 15 U/kg every 24 h for 1 wk • **No TMAs or TEs reported**
US FAERS case report ID 21501134 [45]	1 bleed in 1 patient • Diverticular hemorrhage	• aPCC 43 U/kg, then 10 doses of rFVIIa 71 μg/kg over 2 d, followed by 7 doses of aPCC 43 U/kg over 5 d (including a cumulative dose of > 100 U/kg within a 24-h period) • **1 TMA reported** a
Surgery		
**STASEY** [14]	3 surgeries in 3 patients	• Minor dental surgery (postoperative bleed): 1 dose of aPCC 109.5 U/kg • Major surgery (arthroplasty): 3 doses of aPCC over 3 d (cumulative dose: 80.9 U/kg) • Major surgery (not specified): 2 doses of aPCC for 1 d (cumulative dose: 50 U/kg) • **No TMAs or TEs reported**
Ebbert et al. [39]	1 surgery in 1 patient	• Major orthopedic surgery (postoperative bleed): alternating doses of rFVIIa 90 μg/kg and aPCC 5000 U every 6 h^c^ for a total of 45 doses • **1 TMA reported** a

aPCC, activated prothrombin complex concentrate; d, day; FAERS, Food and Drug Administration Adverse Event Reporting System; h, hour; mo, month; rFVIIa, recombinant activated factor VII; TE, thrombotic event; TMA, thrombotic microangiopathy; wk, week.

a Details of the TE and TMA cases are provided in Supplementary Table.

b This patient was enrolled in HAVEN 1.

c Patient weight unavailable; dose is listed per the case report. The aPCC dose was considered to be > 100 U/kg/day for ≥ 24 h in the emicizumab postmarketing safety report [46].

### 3.1 Management of breakthrough bleeding: clinical trial data

The HAVEN clinical trial program provides the largest amount of published data on the use of BPAs in people with hemophilia A with inhibitors receiving emicizumab prophylaxis [[20], [21], [22], [23]]. Although these studies were not primarily designed to investigate BPA efficacy, it is reflected in the number of doses and duration of treatment required. Data on the number of aPCC infusions used for the treatment of breakthrough bleeds are published for HAVEN 1 and HAVEN 2 [20,21]. Of 104 adults and adolescents who received emicizumab in HAVEN 1, 28 (27%) were treated with aPCC. For most of these patients (24/28 [86%]), no single infusion of aPCC exceeded 100 U/kg, and the median (minimum-maximum) number of infusions was 3 (1-27). In HAVEN 2, 2 children receiving emicizumab prophylaxis were treated with aPCC for breakthrough bleed management, each at a single dose of < 50 U/kg [21]. These findings are consistent with historical clinical data in people with hemophilia with inhibitors not receiving emicizumab prophylaxis, which indicate that most bleeds can be treated with 1-2 infusions of aPCC [[47], [48], [49]].

In a pooled analysis of data from the HAVEN 1-4 trials, 130 bleeds in 37 people with hemophilia A with inhibitors receiving emicizumab prophylaxis were treated with aPCC [22]. The majority of bleeds (105/130 [81%]) were treated with aPCC for ≤ 24 hours, with no TMAs or TEs observed, regardless of dosing. Most bleeds (99/130 [76%]) were treated with ≤ 100 U/kg/day of aPCC, with no TMAs or TEs observed, regardless of treatment duration. More specifically, 87 bleeds (67%) were treated with a cumulative dose of 50-100 U/kg/day of aPCC and 12 bleeds (9%) with a cumulative dose of < 50 U/kg/day of aPCC. The remaining 31 bleeds (24%) were treated with a cumulative dose of > 100 U/kg/day of aPCC, of which 18 were treated for < 24 hours, 3 for 24-48 hours, and 10 for > 48 hours. Two TEs (superficial thrombophlebitis and cavernous sinus thrombosis) were observed in patients who received > 100 U/kg/day of aPCC (1 for 24-48 hours and 1 for > 48 hours) [20,22]. TMAs were reported in 3 of 10 instances in which aPCC was used at > 100 U/kg/day for > 48 hours. Two of the patients who experienced TMAs were also treated with rFVIIa (eptacog alfa) for the same bleeding event [20,22,29,30]. A summary of the TE and TMA cases is presented in the Supplementary Table.

In addition to the data from the HAVEN clinical trials, the STASEY study has provided complementary data on the use of aPCC in people with hemophilia A with inhibitors receiving emicizumab prophylaxis [26]. Overall, 13 bleeds in 5 patients were treated with aPCC, with no TMAs or TEs observed in any of these patients [26].

### 3.2 Management of breakthrough bleeding: RWE and case reports

Limited published RWE is available on the use of aPCC in people with hemophilia A with inhibitors receiving emicizumab prophylaxis. No TMAs or TEs were reported in the 3 case reports (5 instances of aPCC treatment in 4 patients) that have been published [[42], [43], [44]]. In the study by Zimowski et al. [42], 3 acute bleeding episodes were observed in 2 pediatric patients, which were initially treated with rFVIIa. Owing to a lack of efficacy/improvement, treatment was switched to low-dose aPCC, after which the bleeds improved. Batsuli et al. [43] reported a case of bleed resolution in an adolescent who had a history of poor response to rFVIIa. The patient had worsening pain and a > 2 g/dL decrease in hemoglobin levels following treatment with rFVIIa (eptacog beta) and was switched to aPCC. Dargaud et al. [44] reported aPCC use in a person receiving emicizumab prophylaxis as part of the HAVEN 1 clinical trial. The dosing regimen was tailored based on previous investigations into the *in vitro* effects of aPCC and emicizumab in this patient, which used a thrombin generation assay.

Based on a postmarketing safety report for emicizumab (up to August 1, 2023), no TMAs or TEs were reported in people with hemophilia A with inhibitors receiving emicizumab prophylaxis who were treated for breakthrough bleeding with aPCC at ≤ 100 U/kg/day [46]. In addition to the 3 TMA events reported in the HAVEN 1 trial (2 of which occurred in patients who were also treated with rFVIIa [eptacog alfa] for the same bleeding event), 2 other TMAs were reported, both in patients who received aPCC at doses of > 100 U/kg/day for ≥ 24 hours [20,22,29,30,39,45,46]. The 2 additional TMAs occurred in an orthopedic surgical case in which sequential therapy with rFVIIa and aPCC was used for the management of a postoperative bleed (see Section 3.3) [39] and in a patient who received both rFVIIa (eptacog alfa) and aPCC for the management of a diverticular hemorrhage [45,46] (Supplementary Table).

### 3.3 Management of surgery

There are limited data regarding the management of surgery with BPAs in people with hemophilia A with inhibitors receiving emicizumab. The available clinical trial data from the HAVEN 1-4 and STASEY trials (data cutoff: October 2018 and January 2021, respectively) indicate that up to 33% of people with hemophilia A with inhibitors receiving emicizumab prophylaxis require surgery, with hemostatic support managed at the discretion of the physician [14,15]. In the STASEY study, instances of perioperative bleed management with aPCC were reported for minor and major surgeries in 3 people with hemophilia A with inhibitors receiving emicizumab [14]. No adverse events (AEs) or thrombotic complications related to BPA treatment were reported in these patients.

An orthopedic surgical case has also been reported by Ebbert et al. [39], in which sequential BPA therapy was used for management of a postoperative bleed in a person with hemophilia A with inhibitors receiving emicizumab, with alternating doses of 90 μg/kg of rFVIIa and 5000 U of aPCC every 6 hours for a total of 45 doses [39]. A TMA was observed in this patient, which, as noted in Section 3.2, was 1 of 5 TMAs reported in patients who received aPCC at doses of > 100 U/kg/day for ≥ 24 hours in the emicizumab postmarketing safety report [39,46].

## 4 Monitoring in Patients Receiving Emicizumab and BPAs

All procoagulant therapies for bleed management in people with hemophilia have the potential to cause thrombotic complications. Historical data have shown that the use of BPAs is associated with a low incidence of thrombotic AEs, reported to be 4-8 events per 100,000 infusions for aPCC and 12 events in 11,121 bleeding episodes for rFVIIa [[50], [51], [52], [53]]. The incidence of thromboembolic AEs per 1000 exposed patient-years is reported to be 15.4 for aPCC and 18.2 for rFVIIa [54]. Cumulative data from clinical trials indicate that the risk of TEs in patients receiving emicizumab alone or combined with either class of BPAs is also relatively low [20,21,[26], [27], [28]]. In total, 406 adverse drug reactions (ADRs) were reported for emicizumab in 2021 in the EudraVigilance database, which collects data on medicines in the European Economic Area [27]. Of these ADRs, 24 (6%) were thrombotic ADRs, 15 (63%) of which were reported in patients receiving emicizumab only. The remaining 9 (37%) were reported in patients receiving concomitant treatment with BPAs and/or FVIII (4 with rFVIIa [eptacog alfa], 1 with aPCC, 1 with both rFVIIa [eptacog alfa] and aPCC, 1 with rFVIIa [eptacog alfa] and FVIII, and 2 with FVIII) [27]. From 2018-2022, 2383 AEs in patients receiving emicizumab were captured in the US Food and Drug Administration Adverse Event Reporting System, of which 97 (4%) were thrombotic AEs [28]. Of these, 33 (34%) occurred in patients receiving rFVIIa (24 with eptacog alfa and 9 with unspecified rFVIIa products) and 14 (14%) occurred in patients receiving aPCC [28].

Several *in vitro* spiking studies have investigated the combined effect of emicizumab and BPAs on thrombin generation. Initial experiments performed in either hemophilia-simulated plasma with a sequence-identical analog of emicizumab and aPCC or plasma from people with hemophilia A with inhibitors receiving emicizumab prophylaxis that was spiked with aPCC suggested a possible synergistic effect of emicizumab and aPCC, resulting in excessive thrombin generation above normal physiological levels [55,56]. The FIX in aPCC was suspected to be the main driver of this *in vitro* effect [55]. Following this, Kizilocak et al. [31] compared the thrombin generation potential in people with hemophilia A with inhibitors receiving emicizumab prophylaxis and escalating doses of aPCC *in vivo* (5-75 U/kg) with that in plasma from the same patients following *in vitro* spiking of aPCC. They found major differences between the *in vitro* and *in vivo* results, with thrombin generation within the normal physiological range in most patients who received *in vivo* aPCC infusions. The effect on thrombin generation was concentration-dependent and showed considerable interpatient variability, and no AEs were observed in the study. These findings suggest that the results of *in vitro* spiking experiments with emicizumab and aPCC may be misleading [31]. It has been proposed that the discrepancy in thrombin generation between the *in vivo* and *in vitro* studies is due to the *in vivo* clearance of the FIXa present in aPCC [57].

Overall, data indicate that the incidence of thrombotic AEs in patients receiving emicizumab and BPAs is low; however, monitoring these patients remains essential. Variability seen in analyses of thrombin generation as well as limited access to thrombin generation assays limit the applicability of these tools in routine clinical practice [2,58]. While thrombin generation assays may be useful in certain situations, adhering to guideline-defined dosing and monitoring for clinical signs of bleed resolution and thrombosis remain the cornerstones of clinical management with BPA therapy in patients receiving emicizumab.

## 5 Implications for Clinical Practice—Key Considerations for the Use of BPAs in Patients Receiving Emicizumab Prophylaxis

### 5.1 Medical considerations

Historically, both classes of BPAs have been used for bleed management in people with hemophilia with inhibitors [59], with multiple treatment options allowing for better treatment individualization and optimized patient care. This remains true today, although current bleed management guidelines prefer the use of rFVIIa over aPCC in patients receiving emicizumab prophylaxis [2,60]. When aPCC is used for the treatment of breakthrough bleeding, guidelines recommend dosing of ≤ 50 U/kg/dose and ≤ 100 U/kg/day [2,13]. This is consistent with the data from clinical trials and RWE presented in this review demonstrating the efficacy and safety of aPCC when used at doses of ≤ 100 U/kg/day in patients receiving emicizumab.

As discussed in Section 4, the thrombotic risks associated with the use of BPAs alone or in combination with emicizumab are low [20,21,[26], [27], [28],[50], [51], [52], [53], [54]], although guideline recommendations for dosing should always be followed. While general risk factors for development of TMA in hemophilia have not been identified, the World Federation of Hemophilia guidelines urge caution with the use of BPAs in patients receiving emicizumab who have risk factors for thrombosis such as past venous thromboembolism, obesity, smoking, chronic infection, and inflammation [2].

Patient response is a key consideration when selecting which class of BPAs to use because the efficacy of aPCC and rFVIIa differs among patients [2,32]. This was demonstrated in the FENOC study, in which 10-44% of patients had discordant responses to aPCC and rFVIIa [32]. In situations in which both classes of BPAs are available, historical patient responses should be considered. Thrombin generation assays and thromboelastography have been shown to predict patient response to BPAs [44,61,62]; however, these are not standardized tools and are unavailable in many clinical settings [2,58]. Clinical characteristics associated with a differential response to BPAs have not been identified, although both BPAs are effective in the majority of patients [32]. If there is a poor treatment response to the chosen agent, it is critical to switch promptly to the other class of BPAs because delayed switching may prolong bleed management, result in bleed escalation, and worsen bleed outcomes [32,43]. If a patient does not respond sufficiently to either aPCC or rFVIIa, the World Federation of Hemophilia guidelines recommend that combination/sequential therapy can be considered in experienced centers and with close monitoring [2].

Bleed type should also be considered in the treatment plan because this may influence the dosing and duration of BPA use. Most bleeding events in people with hemophilia (70-80%) are joint bleeds, which typically require a shorter treatment duration than muscle bleeds or surgeries [2,43].

### 5.2 Practical considerations

The accessibility and availability of each class of BPAs is an important factor for bleed management in people with inhibitors receiving prophylaxis with NFTs. In resource-constrained countries, physicians and patients may only have (potentially limited) access to specific treatment options [1,2,63]. It is therefore essential that physicians and patients understand how both classes of BPAs can be used optimally for bleed management. Additional factors that should be considered when selecting which class of BPAs to use include patient or caregiver preference, burden of administration, and economic considerations [2].

## 6 Use of aPCC in Patients Receiving Other NFTs

Published rates of breakthrough bleeding reported in clinical trials of the rebalancing therapies concizumab (anti-TFPI monoclonal antibody) and fitusiran (antithrombin-targeting small-interfering RNA) are similar to those seen with emicizumab (Table 4) [16,17,64]. BASIS (NCT03938792) is an ongoing study of marstacimab (anti-TFPI monoclonal antibody), although data on breakthrough bleeding are not yet available [65,66]. It is also noteworthy that trials are in progress for the FVIII-mimicking agents Mim8 and NXT007 (NCT05053139 and NCT05987449, respectively) [4,67,68].

**Table 4:** Rates of breakthrough bleeding in people with hemophilia A with inhibitors receiving prophylaxis with other nonfactor therapies in clinical trials.

Study	NFT	Patient population^a^	Patients with treated bleeds, *n*/*N* (%)^b^	Patients with any bleeds, *n*/*N* (%)^b^
explorer7 (NCT04083781) [64]	Concizumab	HA with inhibitors	43/76 (57)	NR
ATLAS-INH (NCT03417102) [17]	Fitusiran	HA or HB with inhibitors	13/38 (34)	NR
ATLAS-PPX (NCT03549871) [16]	Fitusiran	HA or HB with inhibitors	4/19 (21)	NR

HA, hemophilia A; HB, hemophilia B; n, number of patients who did not experience (treated or any) zero bleeds; N, number of patients in the study-specific population; NFT, nonfactor therapy; NR, not reported.

a Patients were adults and adolescents (aged ≥ 12 years). In the ATLAS studies, data for people with HA with inhibitors were reported together with those with HB with inhibitors.

b Rates of breakthrough bleeding were calculated from the number of patients in the study-specific population who received NFT prophylaxis and did not experience zero bleeds (treated or any, respectively).

Compared with emicizumab, more limited data are available on the use of aPCC in clinical trials of rebalancing therapies, which included people with hemophilia A or B (Table 5). No TMAs or TEs were reported in the patients who received aPCC in these studies [[16], [17], [18]]. In the explorer7 study, breakthrough bleeds in people receiving concizumab prophylaxis could be managed with aPCC (protocol dosing guideline: ≤ 50 U/kg/dose and ≤ 100 U/kg/day), rFVIIa, or a combined product of plasma-derived FVIIa and FX [18]. A total of 45 bleeds in people with hemophilia A with inhibitors were treated with aPCC during the study, most of which (43/45) were mild or moderate in severity; for these bleeds, the median dose of aPCC per infusion was 50 U/kg [64]. In the ATLAS-INH study in people with hemophilia A or B with inhibitors, 2 patients receiving fitusiran prophylaxis were treated with aPCC (protocol dosing guideline: ≤ 50 U/kg/dose, not repeated within 24 hours; recommended single dose of 30 U/kg) [17]. In the fitusiran ATLAS-PPX study, 5 bleeds were treated with 7 doses of aPCC (mean dose: 34 U/kg) [16].

**Table 5:** Use of activated prothrombin complex concentrate for the management of breakthrough bleeding in people with hemophilia A with inhibitors receiving prophylaxis with other nonfactor therapies in clinical trials.

Study	NFT	No. of patients and bleeding episodes treated with aPCC	Dosing summary and thrombotic complications
**explorer7** [64]^a^	Concizumab	45 bleeds^b^	• 43/45 mild/moderate bleeds o Median aPCC dose per infusion: 50 U/kg o Bleed treated with ≥ 3 infusions: 31/43 (72%) • 2/45 severe bleeds o Median aPCC dose per infusion: 80 U/kg o Bleeds treated with 1 and 2 infusions, respectively • **No TMAs or TEs reported** [18]
**ATLAS-INH** [17]^c^	Fitusiran	2 patients	• All bleeds treated in compliance with protocol breakthrough bleed management guidelines^d^ • **No TMAs or TEs reported**
**ATLAS-PPX** [16]^c^	Fitusiran	6 bleeds in 3 patients	• 4/6 treated with a single dose of aPCC o All compliant with protocol breakthrough bleed management guidelines^d^ • 2/6 treated with repeat doses of aPCC o 1/2 compliant with protocol breakthrough bleed management guidelines^d^ • **No TMAs or TEs reported**

aPCC, activated prothrombin complex concentrate; NFT, nonfactor therapy; TE, thrombotic event; TMA, thrombotic microangiopathy.

a Once introduced, breakthrough bleed management guidelines for aPCC in this study were ≤ 50 U/kg/dose and < 100 U/kg/day; the time between the first and second dose was not to be shorter than stated in the local product label [18].

b One mild/moderate bleed in a person with hemophilia B with inhibitors was also treated with a single infusion of aPCC (50 U/kg) [64].

c Data for ATLAS-INH and ATLAS-PPX may include people with hemophilia B with inhibitors [16,17].

d Breakthrough bleed management guidelines for aPCC in these studies were ≤ 50 U/kg/dose, not repeated within 24 hours; recommended single dose of 30 U/kg [16,17].

More evidence is needed on the use of BPAs in patients receiving rebalancing therapies to inform clinical management and BPA choice, although initial results from *in vivo* and *ex vivo* studies suggest similar effects of aPCC and rFVIIa on thrombin generation when combined with concizumab or fitusiran [69,70].

## 7 Conclusions and Future Perspectives

Overall, clinical trial data and RWE demonstrate that people with hemophilia A with inhibitors receiving prophylaxis with emicizumab experience breakthrough bleeding that requires treatment with BPAs (aPCC or rFVIIa) to achieve hemostasis [2]. Access to both classes of BPAs is needed to support individualized bleed management in these patients [2]. The data presented in this review support the efficacy and safety of aPCC at doses of ≤ 100 U/kg/day in patients receiving emicizumab [20,22], consistent with guideline-recommended dosing. Nevertheless, it is critical that the medical community continues to report data on BPA use in patients receiving emicizumab. To date, data have often only been reported for overall patient populations, highlighting an unmet need to capture more detailed, individual patient data for use in comprehensive analyses. Additional evidence is also required for patients receiving other NFTs, to understand how both classes of BPAs can be used optimally for bleed management in these settings, including how different NFT modes of action may affect the dose and efficacy of the BPA used.
