# Supplementary material for: Activated prothrombin complex concentrate in patients receiving emicizumab prophylaxis: from evidence to clinical practice
Source: Res Pract Thromb Haemost. 2025 Jun 17;9(4):102926. doi: 10.1016/j.rpth.2025.102926 (PMC12346059; doi:10.1016/j.rpth.2025.102926)
Supplement: Supplementary Material [file mmc1.docx]

**SUPPLEMENTARY INFORMATION**

**Supplementary Results**

**Supplementary Table S1.** Summary of thrombotic complications reported in people with congenital hemophilia A with inhibitors receiving emicizumab prophylaxis and aPCC.

| **TMA/TE case** | **Type of bleed(s)/surgery** | **Treated with aPCC >100U/kg/day for ≥24 hours?** | **Treated with both aPCC and rFVIIa?** | **Patient information and dosing summary** |
| --- | --- | --- | --- | --- |
| **TMA #1  [1-3]** | - 2 joint bleeds (1 spontaneous, 1 traumatic) - 1 right lower back bleed | **Yes** | **Yes** | **Patient information**   - Age: 36 years - Comorbidities: hemophilic arthropathy, hypertension - Medical history: NR   **Dosing**   - 2 doses of aPCC 94 U/kg over 2 days - 2 doses of rFVIIa 85 μg/kg (2.5 hours apart; cumulative daily dose: 170 μg/kg) - 2 doses of aPCC 94 U/kg (cumulative daily dose: 188 U/kg) - **TMA reported 3 days after treatment initiation** |
| **TMA #2  [1, 2, 4]** | - Joint bleed (traumatic) | **Yes** | **No** | **Patient information**   - Age: 13 years - Comorbidities: asthma (stable, mild), chronic otitis media (serous), Down’s syndrome - Medical history: NR   **Dosing**   - 5 doses of aPCC 74 U/kg over 49 hours - **TMA reported 4 days after treatment initiation** |
| **TMA #3 [1, 2, 5]** | - Rectal hemorrhage | **Yes** | **Yes** | **Patient information**   - Age: 42 years - Comorbidities: hemophilic arthropathy, hepatitis, hypertension - Medical history: ileostomy due to perforated bowel secondary to high-dose opiate use   **Dosing**   - 11 doses of rFVIIa 87 μg/kg over 3 days   - 5 doses of rFVIIa on the same day (cumulative daily dose: 435 μg/kg)   - On this day, 3 doses of aPCC (1 dose of 98 U/kg followed by 2 doses of 65 U/kg; cumulative daily dose: 228 U/kg) - 9 doses of aPCC 65 U/kg over 3 consecutive days - **TMA reported 4 days after treatment initiation** |
| **TMA #4  [6, 7]** | - Postoperative bleed | **Yes^a^** | **Yes** | **Patient information**   - Age: 28 years - Comorbidities: arthropathy - Medical history: hemorrhage - Surgical history: hip arthroplasty   **Dosing**   - Alternating doses every 6 hours of rFVIIa 90 µg/kg and aPCC 5000 U (patient weight unavailable; dose listed per case report; cumulative daily dose: 20,000 U) for a total of 45 doses - **TMA reported (days after treatment initiation NR)** |
| **TMA #5  [8]** | - Diverticular hemorrhage | **Yes** | **Yes** | **Patient information**   - Age: 67 years - Comorbidities: hemophilic arthropathy - Medical history: NR   **Dosing**   - 1 dose of aPCC 43 U/kg - 10 doses of rFVIIa 71 μg/kg over 2 consecutive days - 7 doses of aPCC 43 U/kg over 5 consecutive days - **TMA reported 6 days after treatment initiation** |
| **TE #1  [1, 2, 9]** | - Joint bleed (spontaneous) - Shin bleed (spontaneous) | **Yes** | **No** | **Patient information**   - Age: 42 years - Comorbidities: cholecystolithiasis, hemophilic arthropathy, hepatitis C, insomnia - Medical history: 3 episodes of gastrointestinal hemorrhage (stomach ulcer)   **Dosing**   - 2 doses of aPCC 101 U/kg over 2 days - **TE (superficial thrombophlebitis) reported 2 days after treatment initiation** |
| **TE #2  [1, 2, 10]** | - Joint bleed (traumatic) | **Yes** | **No** | **Patient information**   - Age: 23 years - Comorbidities: anemia, arthralgia, arthropathy, drug hypersensitivity - Medical history: appendicitis, bacteremia, gastric ulcer hemorrhage, hemarthrosis, peptic ulcer disease   **Dosing**   - 8 doses of aPCC over 4 days   - 1 dose 83 U/kg   - 3 doses 86 U/kg   - 2 doses 86 U/kg   - 1 dose 104 U/kg   - 1 dose 87 U/kg - **TE (cavernous sinus thrombosis) reported 3 days after treatment initiation** |

aPCC, activated prothrombin complex concentrate; FAERS, FDA Adverse Event Reporting System; NR, not reported; rFVIIa, recombinant activated factor VII; TE, thrombotic event; TMA, thrombotic microangiopathy.

^a^The aPCC dose was considered to be >100 U/kg/day for ≥24 hours in the emicizumab post-marketing safety report [11].

**References**

[1] Genentech, a member of the Roche Group. HEMLIBRA USA prescribing information; <https://www.gene.com/download/pdf/hemlibra_prescribing.pdf>; 2024 [accessed April 11, 2025].

[2] Oldenburg J, Mahlangu JN, Kim B, Schmitt C, Callaghan MU, Young G, et al. Emicizumab prophylaxis in hemophilia A with inhibitors. *N Engl J Med*. 2017;377:809–18. <https://doi.org/10.1056/NEJMoa1703068>.

[3] US Food and Drug Administration. FDA Adverse Event Reporting System (FAERS). Case ID: 15108306; 2016 [accessed November 21, 2018].

[4] US Food and Drug Administration. FDA Adverse Event Reporting System (FAERS). Case ID: 13479758; 2016 [accessed February 16, 2018].

[5] US Food and Drug Administration. FDA Adverse Event Reporting System (FAERS). Case ID: 16202702; 2017 [accessed March 12, 2024].

[6] Ebbert PT, Xavier F, Seaman CD and Ragni MV. Emicizumab prophylaxis in patients with haemophilia A with and without inhibitors. *Haemophilia*. 2019;26:41–6. <https://doi.org/10.1111/hae.13877>.

[7] US Food and Drug Administration. FDA Adverse Event Reporting System (FAERS). Case ID: 15615885; 2018 [accessed March 12, 2024].

[8] US Food and Drug Administration. FDA Adverse Event Reporting System (FAERS). Case ID: 21501134; 2022 [accessed February 27, 2024].

[9] US Food and Drug Administration. FDA Adverse Event Reporting System (FAERS). Case ID: 13479556; 2016 [accessed February 16, 2018].

[10] US Food and Drug Administration. FDA Adverse Event Reporting System (FAERS). Case ID: 13479646; 2016 [accessed February 16, 2018].

[11] Sarouei K, Barlera S, Polito L, Tobaruela G and Biondo J. Emicizumab prophylaxis in people with haemophilia A: summary of 10 years of safety data on thromboembolic events and thrombotic microangiopathy. *Haemophilia*. 2024;30 Suppl 1:98. <https://doi.org/10.1111/hae.14919>.
